# The Medical Profession

**Published:** 1856-08

**Authors:** 


### EDITORIAL DEPARTMENT.

[The following article, written by us for Putnam's Monthly, some three years since, has been somewhat extensively republished in the medical journals of the country. In looking over it we do not find that our views have undergone any change since its first publication, and as it has never appeared in these pages we have determined to give our readers the benefit of whatever truths they may derive from it. It presents, briefly, our own opinions upon the relations of the medical profession to the people.]

### *The Medical Profession.*

" Vita brevis, ars longa, momentum urgens, experientia fallax, judicium difficilè."

HIPPOCRATIS APHORISMI.

Some time ago, a writer in Putnam's Monthly chose "Doctors" for his theme. He portrayed, not so much a body of men, as individuals of a class; describing faithfully the peculiarities incident to single members, but touching not those generic distinctions, which separate the doctor *par excellence*, the "regular" physician, from the bevy of empirical men of healing, who compass him about on every side.

Now be it known, that we, of this present writing, are a "regular" physician of the straitest sect, and that in our function as editor of a medical monthly, we are noted for orthodoxy immaculate; yielding not an inch to the pretensions of the hosts of quackery. We do not often find occasion to denounce empiricism in the pages of the before-mentioned periodical. Our readers are themselves orthodox; and we do not choose to waste our logic, without a prospect of its reaching the heart of some transgressor. When, however, the occasion arises, we are "enough for it." Homœopaths, Hydropaths, Eclectics, Thomsonians, Chronothermalists, Eidopaths, and the smaller fry of cancer doctors, and nostrum venders, are alike *anathema maranatha.* So strictly conservative are we, that we hold all these various forms of medical art to be arrant quackery; and we will not even in the remotest and most indirect manner, hold fellowship with any of them.

Our object in thus "defining our position," is to draw upon ourselves the necessity of defending it; and to open the eyes of our readers to our true relations, and to the good and sufficient reasons we have for claiming an infinite superiority over all the heresies just enumerated.

All these are quackery—you, my dear sir, who are acquiring new health and vigor from the wet sheet baptism of the hydropath, are doing so in an unconstitutional manner; and the very influence your cure may have upon others, is a seductive inducement to wrong-doing. You, too, my transcendental sister, placing upon the ruby tip of your tongue that infinitesimal pellet, and solemnly attributing the sensations of many succeeding hours to the nonentity you have swallowed, are a sinner for that very act! Think not to shield yourself behind the cloak of your medical adviser! The thing is either right or wrong. That it is wrong, we propose to prove by most conclusive logic. Prepare yourself for conviction! Leave unlocked the gateways of your intellect, while we march in to take possession; armed *cap-a-pie* with argument. Reasoning by induction, by analogy, by synthesis, by exclusion—by reference to experimentation and the rules necessary to success therein—by entreaty, and appeal, and ridicule—by denunciation, and *argumentum ad hominem*—all is fair in this war; so "up guards and at them!"

We have made some stout assertions. In their support we start with this proposition. *Any exclusive system of medicine is necessarily erroneous.*

And here we can imagine a mighty concourse of sisters (male and female in petticoats and trowsers both, but still all generically sisters) uplifting their myriad hands in uni-voiced dissent. They adduce cases without number to the contrary. Oh, yes, good friends! certainly! we admit all your statements. We have not the least doubt, that the pious and Reverend Mr. Silvertongue *did*, by process of parboiling, recover from his bronchitis. He washed not seven times only, but seventy times seven, in the Jordan of hydropathy; and was healed. (Query. Is the fact that the prophet Elisha was the first to prescribe hydropathy—*vide* case of Naaman the leper—the reason why our clerical friends take so like ducks to the water? or do they live such unsexed and effeminate lives, that their systems feel peculiarly the need of this kindly tonic?) And you, too, Mrs. Newlight; undoubtedly your charming infant did survive the measles under homœopathy. We admit your assertions. Don't, madam, bring up that lusty, hair pulling baby in testimony! We see it is alive and breathing, and acknowledge the whole force of the argument.

But we ask most solemnly, what of it? In days of old, when medicine had no existence save as a mysterious element in the sacerdotal function; when men lived or died as God willed, without the luxury of a doctor, or the agony of his bill; people lived about as long as they do now—children had measles, and were tucked away in a corner, to recover by the unaided powers of nature. Strong men lay down with fevers; and lived, or died, according as their time had come or not. Medication is not the most essential element of cure.

Disease is self-limited. Its tendency, in nineteen out of twenty cases, is toward recovery; and that, uninfluenced as to the ultimate result of death or recovery (more or less complete), by any medical interference; unless, indeed, the latter should be murderously severe.

A judicious physician will rarely say that he has *cured* a patient. The patient regained his health truly, but that medical observer who has a true insight into the laws of disease, knows that in all probability he would have recovered unaided. Medical philosophers have a just horror of the *post hoc ergo propter hoc* reasoning. The laws of evidence should apply as strictly to a medical observation, as to a trial for a capital crime. Nothing should be assumed. The true physician guesses at nothing. He analyses all facts before him. By reading and long practice he learns to attribute to symptoms their true value. We say he guesses at nothing. If, therefore, any impenetrable veil is thrown over a case, rendering it too obscure for his acumen, he "waits for developments." He never acts upon an uncertainty, never administers a drug, without a distinct idea of the effect to be produced, and its bearing upon the disease. If he cannot do this, he should do nothing, or resort to the common resource of "calling counsel."

Now, my dear Mrs. Newlight, it does not follow, because your interesting child took sugar pills antecedent to a recovery from the measles, that the pills had any thing to do with its cure. Most intelligent physicians prescribe nothing for this disease, save the recumbent posture and light diet. And so of the greater number of the offspring of Pandora's box. They are themselves curative, and need only the guiding hand of the physician, without his drugs.

Why, then, employ the doctor? Why draw down upon yourselves the phlebotomy of his bill? Because you are unskilled, you cannot estimate the importance of a symptom. The case that is trivial to-day, may be moribund to-morrow. Call your physician, then, not as an apothecary anxious to sell his drugs, but as an observer, careful to note, and quick to appreciate; forewarned and forearmed, and ready to meet the first indication of danger with cautious skill, or Napoleonic energy, as the case demands.

But we are blowing our trumpet in self-glorification, unmindful of the Jerichoan walls to be demolished. Place, then, gaunt quackery in the pillory, and send home your missiles of argument!

What is quackery? That which pretends to more than it can accomplish. Now without descending to details, without illustrating by this or that disease, and without resort to professional technicalities, we propose to show the impossibility of any exclusive system in medicine. A "system" supposes that all the laws of disease have been investigated and ascertained; that some great principles have been evolved, which are alike applicable to all phenomena; that we have unerring powers of diagnosis, and can locate with certainty a disease, and predicate positively its character and tendencies; that we have certain remedies, whose qualities are well known, and whose effects can be unfailingly anticipated; and, finally, that we are fully aware of all the influences, climatic, dietetic, pertaining to regimen, mental, constitutional, and medicinal, which are acting upon our patient. Now not one of all these necessary precursors of certainty and system is, in the full sense of the word, attainable. Disease is changeable — it has, to use a trite allusion, a Protean shape. It is subject to unknown influences, geological, topographical, and epidemic, which we cannot estimate.

All this is in accordance with the manifestations of God's will to man. For His own all-wise purposes, He has implanted within us the seeds of decay, which must, and will germinate. So it is that medicine is ever reaching for that beyond its grasp; ever stretching its eager hand toward the impossible; surmounting one difficulty to encounter another; but yet, not walking in a circle; neither defeated nor discouraged; ever progressing, and acquiring new control over human suffering, but ever as it seems to attain its long sought certainty, like the fruit which Tantalus beheld, it evades our grasp. Therefore it is that medicine can have no system, for system implies certainty.

What then shall it do? Shall it sit with folded hands and see the tide of human suffering roll by — upturned, imploring faces of strong men, and mothers and gentle babes, shrieking for help, unanswered — calling for Lethe, but in vain?

Not so! For if we have not a system, we have at least a museum of facts vast and venerable with time, in which the

"Clustering ages blend their common toil,"

and to which unwearied modern effort is constantly adding. Wo have the knowledge of anatomy; we have an imperfect, but still valuable physiology; teach the student these; tell him all we know about disease; teach him to distinguish one from another; spread before him our vast *materia medica;* tell him its qualities, and its varied control over different morbid actions; and then send him into the world of sickness, to wield these weapons cautiously.

And here is the great point of distinction between the regular profession and the systemists. The latter are men of one idea. Let us instance. Homœopathy says, that like cures like; that the less the cause, the greater the effect. Hydropathy points to the clear running brook, and the health-giving fountain, as the only remedy for disease. The Thomsonian and the Eclectic show us the vast array of the vegetable materia medica, and say, "These alone are our weapons." The chronothermalist insists upon periodicity as the great first principle of all diseases; and the nostrum vender offers his panacea with the reckless assurance that it is always safe.

Wherein do we differ from these sects? Legitimate medicine refuses to take the position of a sect, and will recognize no system, until the necessary knowledge for its formation is attained. The time has not come, in all probability it never will come, when we can have general principles in medicine. For a principle must have no exceptions, and so long as an exception exists, the principle is erroneous and unsafe. Common consent has given to a *rule* the privilege of *occasional* error, if it is only *generally* right; though wherein lies the philosophy of the motto *exceptio probat regulum*, we could never discover. To each of these misnamed systems we return such answers as these: We say to the homœopath, that if like cures like, so also do contrary causes produce cures. The coincidence on either side is equal. To be a principle in medicine, your degree should know no exception, for the first one may cost a life, or, in the sequence of chances, a hundred lives in succession. Hence the danger and absurdity of devotion to a theory. * * * The infinitesimal notion is one to which reasoning cannot apply. The strongest argument against it is this—it is contradictory of an axiom; of the self-evident truth, that an effect will be great in proportion to the greatness of its cause. This is what is called a first truth, an innate, or transcendental idea, which has the force of higher law, and overrules all seeming contradictions.

Against the pluvious doctrines of the hydropath, legitimacy indues itself in india-rubber garments; and thus fortified, it assures him of the distinguished consideration in which it holds water as a beverage, and a remedy. Both uses are valuable; but shall we, because water is a curative, reject all other remedies? It is an ancient and well-approved curative. We have before us a certain black-letter tome, in which one Nicholas Cyrillus, who flourished in Naples at the close of the seventeenth century, treats learnedly *De usu Frigidae, et Frigidorum* in Frebribus;" and in best apothecaries' Latin, discourses "*apud liberaliorem usum de aquam*" in fevers. Water is one of our most cherished weapons, stolen from our armory by the peasant Preissnitz. But Preissnitz, like a child with a new toy, applied it to all manner of illogical and contradictory uses. It is a good thing, but shall we therefore put ourselves to soak like so much dirty linen?

To the Thomsonian and eclectic—*par nobile fratrum*, the same with a difference—it argues thus: You have some good notions, my friends, and you mean that the world shall appreciate their merits; but what have you that we have not? Did you, oh wisest of Onondaga farmers, invent the vapor bath? Was lobelia an unknown weed, until you upset your stomach with it? You are certainly (but purely accidentally) the nearest right of all exclusive schools, but why confine yourself to one arm of the service? It is very well to have different forces, but in a well-conducted war, we need horse, foot, and dragoons. Stick valiantly, then, to steam and lobelia; but cast not aside, as useless, those other and gentler means, provided to our use.

*Jam satis.* Here is our ground. Legitimate medicine demands the whole domain of Nature, in which to seek its remedies. The myriad forms of vegetation, the peculiar products of the animal kingdom, all the potent revelations which chemistry has tortured from the thick-ribbed earth and its minerals; air, fire, and water; diet, regimen, ventilation, mental stimulants, and mental anæsthetics,—all are its remedies. We will allow no theory to forbid their use, and when the private judgment of the individual practitioner dictates the exhibition of any one of them, no *system* should prevent it. The private judgment of the individual is the only safe criterion, and he should be answerable only to his own sense of right and wrong. This is the soul of our code of ethics. All that our associations and authoritative conventions demand, is that each should concede to others this liberty of opinion. Such is the republic of medicine—truest of all republics, holding perfect individual freedom consistent with the safety of all.

Permit us, patient reader, if not already wearied with "much physic," to tell you, *ex cathedra*, what you should expect from your medical adviser. A perfect physician—one competent to all emergencies—is an impossibility. Hence the frequent necessity for consultations. The labor of centuries (were so long a term conceded to one human life) would be insufficient to fill the memory with all the facts having a direct or indirect bearing upon our art, and necessary to its highest attainment. But this does not imply that you have not a very good physician next door. He, who, having many facts, has also a judgment capable of applying them, is the safest man to employ. Choose, therefore, one sensible and honest, whose discretion you would trust in ordinary matters; not given to bestriding hobbies, moderate in opinion, quick in perception, and calm in danger. Let this man be habitually studious, and you will not go amiss. Such a man will deal honestly with you, will inform you of probabilities as they appear, and dangers as they arise; and when his skill has eased your sufferings, has given sleep to your pillow, or composure to your mind; when it has (as it not unfrequently may) rescued you from death itself, or from the wretched sequences of imperfect cure, then pay him liberally. As you value your life, shun a cheap physician. The old proverb applies—"*Cheap and nasty*," and is as true of doctors as of dry goods.

We take it for granted that we are addressing generous souls. To secure the presence of talent in a profession, its services should have a high cash value. But no other profession performs so much unrewarded labor; no craft displays so wide a charity. In nearly all of our dispensaries, and in very many hospitals, the larger amount of medical service is performed gratuitously; and it is from individual labor alone that the physician can acquire wealth. He can delegate no portion of his functions to clerks or assistants. This is ever to us a painful subject for reflection; and from our inmost heart goes up the groaning supplication, *Eheu! Quamdiu Domine!*

It is (and we thank God for the knowledge of a truth which gives us faith in human nature)—it is our privilege to know many men—physicians in the deep obscurity of country villages, or in the deeper sol tude of cities—men great in mind and in attainment, pure in life and charitable in heart, contented with the oblivion which surrounds them, in the consciousness of fulfilled duty, hiding under a napkin those talents which might shine gloriously among men, yet destined to hear, "Well done, good and faithful servant!" from the Lord of the vineyard. We know men of talents, which in trade would make them merchant princes; in law would bring them honor and distinction; or who in literature would rank as stars; toiling night and day without sleep, or that Sabbath rest which we deny not to the beast of burden, for a pittance of support, unworthy of a steamboat-runner's talents.

Toil on, oh faithful hearts, by fever-beds, and rank contagion in the hovels of the poor! Better than gold the consciousness of doing good!

